# Adolescent - parent communication on sexual and reproductive health issues among high school students in Dire Dawa, Eastern Ethiopia: a cross sectional study

**DOI:** 10.1186/1742-4755-11-77

**Published:** 2014-11-07

**Authors:** Mulatuwa Ayalew, Bezatu Mengistie, Agumasie Semahegn

**Affiliations:** College of Health and Medical Sciences, Haramaya University, Po. Box- 235, Harar, Ethiopia

**Keywords:** Communication, Sexual, Reproductive, Adolescent, Parent, Ethiopia

## Abstract

**Background:**

Sexual and reproductive health communications are most likely promoting healthy sexual development and reduce sexual risks. Communication is the principal means for parents to transmit sexual values, beliefs, expectations and knowledge to their adolescents. However, there is a paucity of evidence about adolescent parent communication in Ethiopia. This study aimed to determine adolescent-parent communication on sexual and reproductive health issues and associated factors among high school students in Dire Dawa, Eastern Ethiopia.

**Methods:**

Institution based cross sectional study was conducted among high school students in Dire Dawa administrative council from February to March 2011. Simple random sampling technique was used to select 695 students from 9–12 grades. Qualitative data were collected through focus group discussion separately for female and male parents. Data were entered in Epi info version 3.5.1 and analyzed by SPSS version 16.1. Logistic regression with OR and 95% confidence interval was used to identify the independent predictors of adolescent parent communication.

**Results:**

Thirty seven percent of students had ever discussed on at least two sexual and reproductive health topics with their parents. Of which, majority of student preferred to discuss with their peers than parent. Condom use during first intercourse was associated with having communication about sexual and reproductive health [AOR = 1.9, 95% CI: 1.0, 3.8]. Cultural taboo, shame and lack of communication skill were reasons that hinder communication between parent and adolescent about sexual matters.

**Conclusion:**

Communication on sexual and reproductive health issue between adolescent and their parent was low. School based education is important to improve adolescent parent communication about sexual and reproductive health issues.

**Electronic supplementary material:**

The online version of this article (doi:10.1186/1742-4755-11-77) contains supplementary material, which is available to authorized users.

## Introduction

World Health Organization defines adolescence and young people are persons whose age between 10–19 years and 10–24 years respectively [1]. There are more than one billion adolescent people worldwide in which seventy percent of them live in developing nations. They are disproportionately affected by HIV that is particularly higher in Sub-Saharan Africa [2]. Sixteen million late adolescent girls give birth every year, in which 95 percent of births occur in developing countries [3]. One fourth of adolescents have sexually experience in East, Southern and West Africa. Parent–child sexuality communication in Sub-Saharan Africa is steadily increasing. Early exposure to sex education by mothers is reporting to encourage early sexual debut. Communication about sexuality is low across countries [4].

Adolescents frequently engage in risky sexual behaviors that adverse health outcome including unintended pregnancy and sexually transmitted diseases. Parent-adolescent communication is important because sexual activities begin at early age for many adolescents [5]. Rates of sexual initiation during young adulthood are rising or remaining unchanged in many developing countries, and high HIV prevalence adds to the risks associated with early sexual activity [2]. Parents have significant potential to reduce sexual risk behaviors and promote healthy adolescent sexual development. One way that parents can realize this potential is through communicating with their adolescents about sexual behaviors and decision-making [6]. School based comprehensive sex education programs more often focus on the delay of sexual activity, training in sexual negotiation, communication skills, information about obtaining contraceptive and reproductive health services. Both abstinence and comprehensive programs aimed at this group tend to focus on puberty, pregnancy, HIV information, assertiveness and refusal skills. Parent-based approaches could be an effective strategy in the repertoire of programs to delay sexual intercourse, reduce teenage pregnancy and sexual transmitted infections [7].

Thirty three percent of Ethiopia population comprises 10–24 years old [8]. Sexual communication is crucial aspect of sexual socialization and fundamental process of parents convey ideas, values, beliefs, expectations, information and knowledge to their children [9, 10]. In Ethiopia, 60% of adolescent pregnancies are unwanted resulting from unprotected sexual intercourse [11]. Among youth whose age between 15–24 years old, 1.1% of women are infected with HIV [12]. According national reproductive health strategy of Ethiopia, few national programs are specifically targeted towards addressing their most pressing reproductive health needs. Eighty four percent of adolescents resides in rural area who have limited access to targeted reproductive health services for young people contributes to, and exacerbates many of the reproductive health problems. Unwanted pregnancies, high abortion, contract sexual transmitted infections including HIV among young people are most likely common because of the risky and non-voluntary nature of their sexual activities [13].

However, sexual and reproductive health service is not inclusive; restricted at health facility level and not evidence based. Sexual and reproductive health service has not been provided at school, at community and at family level. Parent-adolescent communication about sexuality is the controversial issue. Most parents do not feel comfortable to talk with their adolescents about sexual issues. They tend to limit conversations to safe topics [7]. There is little information about level of parent-adolescent communication and associated factors in the region.

Adolescents and youths are the most risky portion of population in the world. They are victim of different avoidable sexual and reproductive health negative consequences such as unwanted pregnancy, unsafe abortion and sexual transmitted infections including HIV/AIDS. Almost all of sexual and reproductive health problems are preventable via transparent discussion, life skill training and making adolescents assertive on sexual and reproductive matters. Accessing sexual and reproductive health service to adolescent and young people helps to avoid many health problems, and achieve the millennium development goal 3, 4, 5 and 6. Determining parent-adolescent communication about sexual and reproductive health issues helps to design appropriate intervention programs. Therefore, this study aimed to assess adolescent-parent communication on sexual and reproductive health issues among high school students in Dire Dawa, Eastern Ethiopia.

## Materials and methods

### Study setting and period

The study was conducted in Dire Dawa administrative council from February to March, 2011. Dire Dawa is a commercial and industrial center located 515 kilometer from Addis Ababa on the Addis Ababa–Djibouti railroad in the eastern part of Ethiopia. Dire Dawa administrative council has estimated area of 128,802 hectares that consists of 9 urban and 38 rural kebeles. Based on the 2007 Central Statistical Agency of Ethiopia, Dire Dawa had population of 341,834, of whom 171,461 were men and 170,461 women. According to the 2010 statistical report of Dire Dawa education bureau, there are 10,234 students attending in eight governmental and eight private high schools.

### Study design and sample size determination

Institution based cross sectional quantitative and qualitative study was employed. Six hundred ninety five in-school adolescents were selected from 9–12 grades in the academic year 2010/11. Sample size was determined using single population proportion formula by considering assumptions of proportion of parent-adolescent communicating on sexual and reproductive health issues assumed to be 28.9% [10], desired precision of 5%, 95% confidence level. Design effect of two plus 10% for non response rate, a total of 695 students were required for the study.

### Sampling procedure

Series of sampling procedures were used to select study subject. First, multi stage sampling procedure was employed to select representative sample of students in the selected high schools. From six schools that have grade 9–12, four high schools (two governments and two private) were randomly selected. The sample size was proportional allocated to each stratum (Grade 9, 10, 11 and12). Among 4350 students, 695 study participants were selected by simple random sampling (lottery method) using the roster as sampling frame. Students who are blind and or seriously sick at the time of data collection were excluded from the study. Purposively selected day-time student parents’ were involved in four focus group discussions to explore community perception about communication on sexual and reproductive health issue. Each focus group discussions composed of 9 to 10 parents.

### Data collection instrument and method

Data were collected using pretested structured self administered questionnaire. The questionnaire was adapted from previous studies and Global School-based Student Health Survey (GSHS), Core-Expanded Questions for the Module on Sexual Behaviors (WHO, 2010). The questionnaire consisted of socio-demographic characteristics and sexual behavior of student. Data collectors were trained for one day on the objectives of the study, sampling procedure, questionnaire, checking the completeness of questionnaire. Confidentiality was maintained by reminding study participants not to write their names and put questionnaires on a table after they have completed. Qualitative data were collected by trained facilitators and principal investigators using open ended interview guides. The responses were tape recorded. The questionnaires were prepared in English and translated local language (Amharic). Then back translated from Amharic to English was made for word meaning consistency by independent body.

### Data processing and statistical analysis

To ensure the quality of the data, all the filled questionnaire were checked for incompleteness and inconsistency. Data were edited, coded and entered using EPI Info version 3.5.1 and then transport to SPSS window version 16.0 for statistical analysis. Descriptive statistical analysis was used to compute frequency, percentage and mean for independent and dependent variables. Binary logistic regression analysis was used to ascertain the association between explanatory variables and outcome. Variables with significant association in the bivariate analysis were entered in to multivariate analysis to determine independent associated factor of adolescent-parent communication on sexual and reproductive health issues. Variables with P value less than 0.05 was considered as significant Qualitative data was transcribed through replaying the tape recorded interview from focus group discussion. The text was thoroughly read and similar ideas bring together. Their inductive meanings were extracted and described in narratives using well said verbatim of participants. The verbatim of participants were transcribed by the three authors independently to confirm the reliability of the finding. The qualitative study findings were triangulated with the quantitative results.

### Ethical consideration

Ethical clearance was obtained from Haramaya University, institutional research ethics review committee. Permission was obtained from school administration and schools parent-teachers committee. Informed verbal consent/assent was obtained from every participant after explaining the purpose of the study in detail. Confidentiality of information was kept anonymously.

## Results

Six hundred ninety five students participated in the study. Six hundred forty one participants completed the questionnaire with a response rate of 92%. Three hundred forty seven (54.1%) and 294(45.9) were male and female, respectively. Ninety one percent of students were between 15–19 years old. The mean age of study participants was 17.1(±1.5) years old. Almost half of students were Amhara in ethnicity. Almost two third of students were orthodox [Table 1]. The majority, seventy percent of students lived with both parents. Four out of every ten students’ family were government employee. Less than one third of students reported their mothers had no formal education [Table 2].

**Table 1:** **Socio demographic characteristics of students in Dire Dawa, Eastern Ethiopia, February – March, 2011 [n = 641]**

Variable	Frequency	Percent
**Sex**		
Male	347	54.1%
Female	294	45.9%
**Age**		
<15	39	6.1%
15-19	581	90.6%
20-24	21	3.3%
**Grade**		
Grade 9	233	36.3%
Grade 10	167	26.1%
Grade 11	126	19.7%
Grade 12	115	17.9%
**Ethnicity**		
Amhara	316	49.3%
Oromo	165	25.7%
Tigre	51	8%
Adere	39	6.1%
Somali	34	5.3%
Others	32	5%
**Religion**		
Orthodox	390	60.8%
Muslim	189	29.5%
Protestant	51	8%
Catholic	7	1.1%
Others	3	0.5%
**Living arrangement**		
With both parent	409	63.8%
With one parent	152	23.7%
With relative	54	8.4%
With friends	13	2%
Alone	13	2%

**Table 2:** **Socio demographic characteristics of the parents of student in Dire Dawa, Eastern Ethiopia, February – March, 2011 [n = 641]**

Variables	Frequency	Percent
Marital status of parents		
Together	452	70.5%
Separated	88	13.7%
Widowed	81	12.6%
Divorced	20	3.1%
Educational status of father		
No formal education	102	15.9%
Primary	92	14.4%
Secondary	180	28.1%
Diploma	93	14.5%
Degree	158	24.6%
Others	13	2%
Educational status of mother		
No formal education	189	29.5%
Primary	141	22.0%
Secondary	182	28.4%
Diploma	61	9.5%
Degree	66	10.3%
Others	2	0.3%
Occupational status of father		
Private	246	38.4%
Government	214	33.4%
Merchant	112	17.5%
Farmers	43	6.7%
Others	21	3.3%
Occupational status of mother		
House wife	280	43.7%
Merchant	128	19.9%
Government	120	18.7%
Private	96	15%
Farmers	9	1.4%
Others	8	1.3%

### Knowledge and communication about sexual transmitted infections

More than three fourth (77.2%) of students knew about common sexual transmitted infections including HIV/AIDS. Almost, half (53.4%) of students knew about HIV/AIDS followed by gonorrhea 214 (33.4%) [Table 3]. Three hundred fifty eight (55.9%) of students discussed about HIV/AIDS, from these, more than half 152(54.1%) of them were discussed with their peers. The reason mentioned by students being ashamed and lack of communication skill were 87(24.2%), 70(19.4%), respectively. Majority of discussant said, “…parents *do not openly discuss about facts of human reproduction with their children. Most of parents stressed avoiding premarital sex to prevent unwanted pregnancy and HIV/AIDS…*”

**Table 3:** **Knowledge of high school students on sexually transmitted infection in Dire Dawa Administrative Council, February - March, 2011**

Variable	Frequency	Percentage
HIV/AIDS	342	53.4%
Gonorrhea	214	33.4%
Syphilis	169	26.4%
Lymphogranuloma venerum	119	18.6%
Chancroid	117	18.3%
Herpes simplex	115	17.9%

### Knowledge and communication about contraceptives

**Figure 1:**
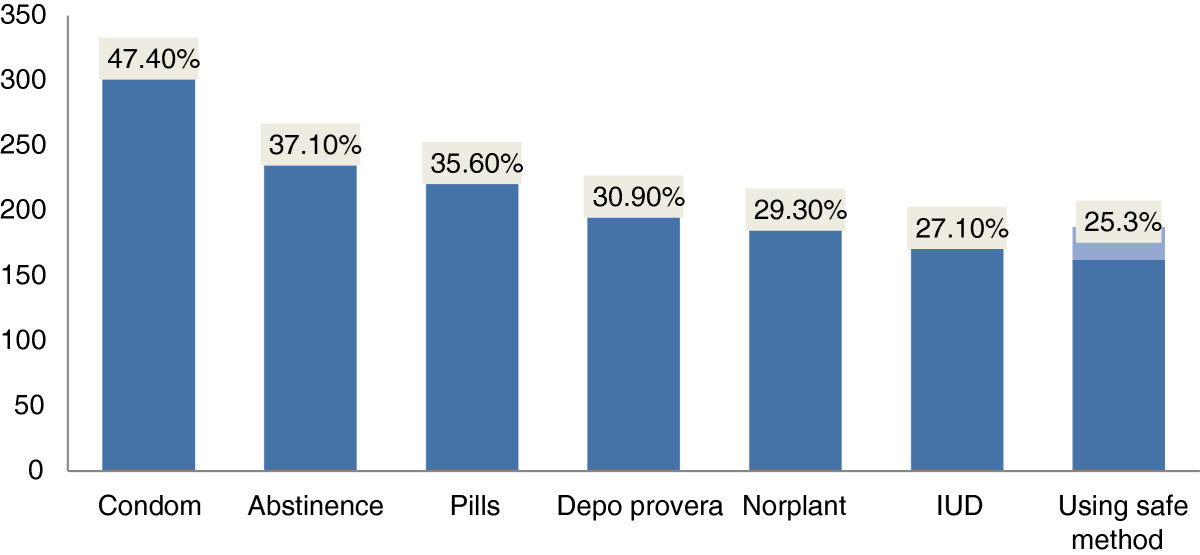
**High School student knowledge on contraceptives in Dire Dawa Administrative Council, February - March, 2011.**

Five hundred thirty one (82.8%) of students knew about at least one contraceptive method that are used to prevent unwanted pregnancy. Condom (47.7%) followed by abstinence (37.1%) were mainly reported contraceptive methods to prevent unwanted pregnancy [Figure 1]. Half of students discussed on contraceptive method mainly with their peers. Two hundred nineteen (34.1%) of study participants discussed about unwanted pregnancy, of these, 124(56.6%) of them discussed with their mothers and with their peers. Almost all focus group discussants were not comfortable to have discussed on sexual and reproductive issues with their adolescents. None of discussants accepted premarital sex. One female participant said, “*I tell her not to happen first but if once she became pregnant, I will give care for her and her child…but… becoming pregnant before marriage brings negative attitude about her in the community and also her family that lead to psychological impact.”*

More than half (53.1%) of students discussed about avoiding premarital sex to prevent unwanted pregnancy, and also one in five in school students (21.7%) had ever discussed about condom use to prevent unwanted pregnancy and sexual transmitted infections. However, lack of communication skill 94(22.3%) and being ashamed 89(21.0%) mentioned as a reason for failing to discuss about contraceptive.

### Sexual attitude and behaviour of students

Approximately one-third (32.3%) student strongly disagreed having premarital sex. Two hundred thirty four (36.5%) of students, strongly agreed about maintain their virginity until marriage. On the other hand, two hundred fifty (39%) of respondents strongly agreed premarital sex if they use condom. The majority, 461 (71.9%) of students heard about sexual and reproductive health issues from mass media, followed by school 290(45.2%), peer 243(37.9%), and family 137(21.4%). One fourth of students were sexually active. The mean age at first intercourse was 15.3(±1.5) years old. Eighty nine sexually active students used condom during sexual intercourse. Of them, sixty four male and twenty five female in-school adolescents used condom during first sexual intercourse. In addition, fifty nine of male students and thirty of female students used other birth control methods.

Regarding to sexual partner history of students, seventy in school adolescents had multiple sexual partner. Sixty nine percent of students disapproved premarital sex. Female students 238(53.6%) more disapproved premarital sex than male students 206(46.3%). Nevertheless, seventy two percent of male students accepted premarital sex. More than one fourth (28.5%) of female students were accept premarital sex. However, the reason of premarital sex disapproval were to maintain their virginity until marriage, religious value, fear of STIs, waiting until getting older and fear of unwanted pregnancy 30.7%, 15.9%, 12.6%, 5.9%, 4.5%, respectively. Almost the entire focused group discussants agreed on girls maintained their virginity until marriage. One male parent said, *“… it is not culturally and religiously acceptable to have premarital sex in the community… and daughter should keep their virginity until marriage….”* Generally, more than half (58.2%) of students had negotiation skill not to have sex with their partner. Of these, female (44.8%) and male (55.2%) of students had negotiation skill not to have sex with partners. Similarly, more than half (57.0%) of male and 43.0% of female students had negotiation skill not to have sex without condom.

### Associated factors for communication on sexual and reproductive health issues

Seventy seven percent of students recognized the importance to discuss about sexual and reproductive health issues with their parents. However, only 36.8% of students had ever discussed at least two sexual and reproductive health issues. The odds of discussing on sexual and reproductive issues were 40% less in males compared to females’ students [OR = 0.6, 95% CI: 0.4, 0.8]. Grade 12 students were 1.6 times more likely to discuss on sexual and reproductive issue with their parent than those in grade nine [OR = 1.6, 95% CI: 1.1, 2.5]. The Odds of discussing on sexual and reproductive health issues is 2.1 times higher among students who have negotiation skill than students who do not have negotiation skill with their partner [OR = 2.1, 95% CI: 1.5, 3.0]. Students who use condom during their first sexual intercourse were 1.9 times more likely to have odds of communication about sexual and reproductive health issues than those who do not use condom [OR = 1.9, 95% CI: 1.2, 3.8] [Table 4].

**Table 4:** **Factors related with discussing on at sexual and reproductive health issues, Dire Dawa, Eastern Ethiopia, 2011**

Variables	Yes (%)	No (%)	COR (95% CI)	Adjusted OR
Sex				
Male	112(47.4)	235(580)	0.6(0.4,0.9)	0.8(0.4,1.7)
Female	124(52.5)	170(42)	1.00	1.00
Grade				
9	73(30.9)	160(39.5)	1.00	1.00
10	68(28.8)	99(24.4)	1.2(0.7,1.9)	1.0(0.3,3.4)
11	46(19.5)	80(19.8)	1.5(0.9,2.2)	0.9(0.4,2.2)
12	49(20.7)	66(16.3)	1.6(1.1,2.5)	1.8(0.9,3.7)
Condom use during first intercourse				
Yes	39(43.8)	50(56.2)	1.9(1.02,3.8)	1.9(1.02,3.8)
No	20(28.2)	51(71.80)	1.00	1.00
Negotiate with partner not to have sex				
Yes	165(44.2)	208(55.8)	2.1(1.5,3.0)	1.0(0.4,2.3)
No	71(26.6)	196(73.4)	1.00	1.00
Negotiate with partner on safer sex				
Yes	161(43.2)	212(56.8)	1.9(1.4,2.7)	1.4(0.6,3.0)
No	74(27.8)	192(72.2)	1.0	

Three-fourth (74.7%) of students preferred their peers to discus about their sexual and reproductive health issues. However, students also discus about their sexual and reproductive health issue with mother, sister, brother and father were 15.4%, 11.4%, 8.9%, 5.9% respectively. But only 17.9% of fathers and 25.4% mothers were transparent and willing to discuss on sexual and reproductive issues. Students were 2.9 times more likely discussed with their mother about sexual and reproductive health issues than other family members. Students were 2.8 times more likely discussed with their brothers and sisters about sexual and reproductive health issues than other family members. Most of mothers usually discussed about menses with daughter. One mother said, “…*I share my experience to my daughter about the precaution she takes when menses come…*” None of male participant discussed about menstruation with their daughter. Fathers most of the time discussed with their son and mothers with daughters due to cultural barriers.

## Discussion

This study determined the status of adolescent-parent communication on sexual and reproductive health issues among in-school adolescents. The findings from this study showed that more than three fourth of students knew about common sexual transmitted infections including the current pandemic HIV/AIDS. Eight out of every ten students knew contraceptive methods to prevent unwanted pregnancy. Students had first sexual intercourse at the mean age of 15 years old. Approximately seven out of every ten student disapproved premarital sexual practice. However, female students were slightly higher than male students disapprove premarital sexual practice. Almost not more than half of students discuss on sexual and reproductive health issues but peer communication is the predominant one. Most of mothers discussed with their adolescents about sexual and reproductive health issues, but none of male participant discussed about menstruation with their daughter. Fathers most of the time discussed with their son and mothers with daughters due to cultural barriers.

This study finding showed that more than one third of students who have communication at least two sexual and reproductive health topics with their parent. This finding is higher than other studies conducted in Benishangul Gumuz in north western Ethiopia and Hawassa in southern Ethiopia [14, 15]. Six out of every ten student ever discussed on HIV/AIDS related issues. This is relatively lower than a finding from study done in Ghana and systematic review study South Africa found that approximately three quarter of students had talked about HIV/AIDS with parents [16, 17]. This is may be due to difference in accessing information and the background of parent. Discussion about sexual and reproductive health issue was associated with condom use. This is consistent with study done in Mexico revealed that those students who had having discussion on sexual and reproductive health issue with parent influence adolescents’ sexual behavior [18]. Discussing about sexual and reproductive health issues and negotiation skill on safer sex significantly associated before controlling for other factors which is similar finding with study done in Atlanta Georgia [19]. This study is almost similar with a study done in Tanzania found that parents did not seem to communicate with their school daughters about sexual and reproductive health issues and the communication was always delivered as general warnings about the negative consequences of premarital sex, HIV/ AIDS, condom use and unwanted pregnancy on their education [20].

In this study, communication about sexual and reproductive health issue with parent has insignificant association with premarital sexual commencement this may suggest that student attitude on acceptance premarital sex mediate involvement in sexual activity. This is contradicting with study done in United States of America [20, 21]. Similarly educational status of parent had no association with having communication about sexual and reproductive health issues. This probably sexual conversation is demanded as taboos which contradict with studies done in Hawassa and Benishangul Gumuz [14, 15]. This study is consistent with a systematic review in Sub-Saharan African countries found that parents reported discussing on human growth and development, pregnancy, childbirth and abortion. Half had discussed sexual transmitted infections, contraception was least discussed. Only 16% of parents had discussed all sexual and reproductive health topics. Mothers were most frequent initiators of discussions. Frequency of communication increased with higher level of education of the parents [4]. This finding contradicted with a study done in East Welloga, Parent young people communication about sexual and reproductive health issues usually initiated by parents and the communication was positively associated with mothers and fathers level of education [22].

This study showed that female adolescent’s students disapproved premarital sex than male students. This finding is inlined with a study done in Kenya found that there were significant difference between male and female towards premarital sex. Males had premarital sex than female adolescents. Adolescents had conservative attitude towards, unwanted pregnancy, induce abortion, contraceptive [23, 24]. Similarly, systematic review in Latin American and Caribbean Literature in parent adolescent sexual communication seemed to be more protective for females than males. It examined the direct association between parent adolescent sexual communication and adolescent sexual and reproductive health outcomes. Overall, there was only sufficient evidence to support a protective association between parent adolescent sexual communication and early sexual debut [25].

Cultural taboos, being ashamed and lack of communication skill of adolescent makes them not to discuss openly with their parent about sexual and reproductive health issue which is similar other studies [14, 15]. This is due to the fact that sexual conversations are deemed a taboo subject in many African communities, for example in Ghana, Sierra Leone, Nigeria and South Africa, this finding is consistent with this study which suggests that parents limit them self to safe topics that students do not discuss about sexual issue with parent [26]. The preference of student to discuss on sexual issues depends on same sex. This is consistent with study done in Hawassa among high school students and study done in China among adolescents where significant gender difference in the pattern of sex communication with parent [14, 27]. This finding is inlined with study done in East Welloga stated that the reason for not discussing about sexual and reproductive health with their parent is fear of parents, embarrassment, taboo attached to sex, parent failure to give time to listen, and parents lack of interest to discussion [2, 22]. The focus group discussion finding of this study also suggests mothers are more comfortable to talk with their daughter and father with son.

### Implication of the finding

Adolescent and youths accounts more than 20 percent of the global population. According to Ethiopian demographic and health survey 2011, one fourth of young women and three out of every ten young men age 15–24 years old had experience sexual intercourse. Although young people are mainly face a lot of reproductive problems, they have been masking by different cultural and religious factors that limits for open discussion on their reproductive health issues. Access to sexual and reproductive health service has contribution to achieve millennium development goal 3, 4, 5 and 6. Multidimensional nature of sexual and reproductive health negative outcomes among young people such as unwanted pregnancy; HIV/AIDS, unsafe abortion and school dropout etc. most of sexual and reproductive health problems are easily avoidable through positive communication and make adolescents assertive on sexual matters. Therefore, assessing adolescent-parent communication on sexual and reproductive health issues and associated factors helps for policy makers, health care providers and any concerned bodies to design appropriate intervention strategies to tackle young generation reproductive health problems. Information obtained here can be used for planning of intervention programs in different part of the country.

### Strength and limitation of the study

The strength of this study is used quantitative and qualitative data presented triangulated. However, it has limitations that it was based on self-reporting and it might be affected by social desirability bias because of sensitive nature and cultural barrier for open discussion. Since the study design was cross section cause and effect relationship could not be established. Analytical study design is recommended for further researches.

## Conclusion

This study finding showed that there were low communication about sexual and reproductive health issues between parent and adolescent. Adolescents discussed about sexual matters more with peers than parent. Condom use during first intercourse was associated with having communication about sexual and reproductive health. Cultural taboo, feel ashamed and lack of communication skill affect adolescent-parent communication on sexual matters. Parents mainly focused on the negative consequence sexual intercourse. Communications about sexual matters depend on same sex basis. Promote parent-adolescent communication on sexuality and improve peer to peer sexuality education program incorporating in to school curriculum, promoting school sexual and reproductive health clubs to enhance parent-adolescent communication and providing information education communication and behavioral change communication materials. Further studies among parents are recommended.

## Authors’ original submitted files for images

Below are the links to the authors’ original submitted files for images. Authors’ original file for figure 1
