# Supplementary figures and images for: Adolescent - parent communication on sexual and reproductive health issues among high school students in Dire Dawa, Eastern Ethiopia: a cross sectional study
Source: Reprod Health. 2014 Nov 7;11:77. doi: 10.1186/1742-4755-11-77 (PMC4233096; doi:10.1186/1742-4755-11-77)

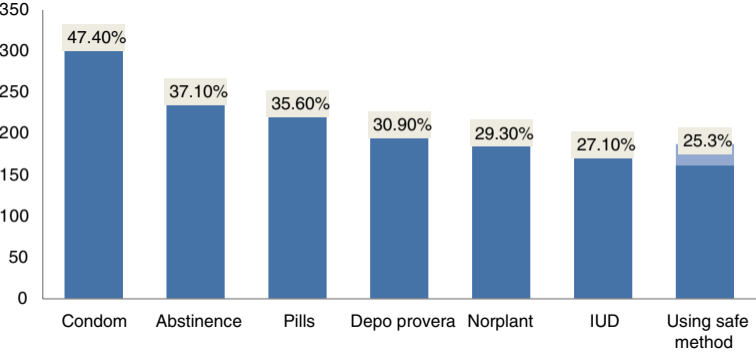

Supplement: Supplementary file 1 — Authors’ original file for figure 1 [file 12978_2013_326_MOESM1_ESM.pdf]
